# Complications associated with loop ileostomy reversal delayed greater than twelve months

**DOI:** 10.1038/s41598-024-74372-x

**Published:** 2024-10-18

**Authors:** Jinman Cai, Madaliene Denison, Hunter Sharp, Mia Edelson, James Kwok, Molly Scarbro, Farrell Adkins

**Affiliations:** 1 Department of Surgery, Carilion Clinic - Virginia Tech Carilion School of Medicine, 1906 Belleview Ave SE, Roanoke, VA 24014 USA; 2 Health Analytics Research, Carilion Clinic, Roanoke, VA 24014 USA

**Keywords:** Gastrointestinal diseases, Intestinal diseases

## Abstract

Diverting loop ileostomy is performed after colectomy to allow for anastomotic healing, and prevention of pelvic sepsis when an anastomotic leak occurs. There is no consensus on the optimal timing of ileostomy closure, and there is limited data on complications associated with ileostomy closure greater than 12 months after creation. The aim of this study is to investigate outcomes of delayed loop ileostomy closure greater than 12 months after creation. Patients undergoing loop ileostomy closure between 2013 and 2023 at Carilion Medical Center, in Roanoke, VA were reviewed. Cohorts compared were defined as Control Group (closure < 4 months) and Delayed Group (closure > 12 months). Demographics and outcomes were compared. Statistical comparisons were performed using either Wilcoxon rank sum test, Pearson’s Chi-squared test or Fisher’s exact test. Statistical modeling included binary logistic regression for 30-day readmissions and a generalized linear modeling for days till bowel function returns. Adjusted odds ratios, confidence intervals, and p-values were calculated. There were 135 patients in the Control Group and 19 patients in the Delayed Group. Demographics were similar between the groups except for a higher percentage of patients with diabetes, renal failure and history of cancer in Delayed Group (all *p* < 0.05). Operative time was longer for Delayed loop ileostomy closure (*p* < 0.05). Patients in the Delayed Group demonstrated a higher hospital readmission rate within 30 days (*p* < 0.05). Both groups had similar return of bowel function on post-operative day 2, similar length of stay, and similar rates of postoperative ileus (p = NS). Delayed loop ileostomy closure more than 12 months after creation does not delay return of bowel function but may lead to higher hospital readmission rates within 30 days.

## Introduction

The practice of creating a diverting loop ileostomy is a well-established intervention employed for various indications, including diverticulitis, inflammatory bowel disease and colorectal malignancies. Loop ileostomy is not only created in colorectal surgery for mitigating the impact of an anastomotic leak, but also as a prophylactic measure in toxic megacolon or as a damage control option in emergency surgery^1,2^. Loop ileostomy closure is generally recommended within 3–6 months after primary operation to achieve sufficient healing while avoiding an extended presence of a loop ileostomy with possible subsequent risk of developing stoma-related complications^3^. Currently there are no established guidelines to define the optimal timing to close a loop ileostomy. Varied perspectives exist, with some advocating for reversal between 6 and 12 weeks after the index operation, while others consider reversals beyond six months as delayed^4–6^. Evidence suggests that delayed ileostomy closure is associated with higher rates of postoperative ileus, delayed recovery of gastrointestinal function, higher rates of small bowel obstruction, increased wound complications, increased risk of Low Anterior Resection Syndrome (LARS) in rectal cancer patients, and longer length of hospital stay^3,5,7–11^.

Outcomes of loop ileostomy closure beyond 12 months after creation are less well defined. In this study, we aim to investigate the outcomes of delayed loop ileostomy closure performed greater than 12 months after creation. A comparative analysis with ileostomy closure conducted within four months after the index operation will be undertaken.

## Materials and methods

### Study population and data collection

This retrospective cohort study was approved by the Institutional Review Board (Clinical Trial Number IRB-22-1813) with IRB-approved waiver of informed consent. All methods were performed in accordance with the relevant guidelines and regulations. This research received no funding. All patients who underwent loop ileostomy closure at Carilion Medical Center in Roanoke, VA between January 1, 2013 to December 31, 2023 were included in the study. No patients were excluded based on diagnosis, which included complicated diverticulitis, colorectal cancer, inflammatory bowel disease and trauma. Patients under the age of 18 years were excluded as were patients undergoing loop ileostomy closure between four and 12 months after index operation. Patients who underwent laparoscopic reversal of loop ileostomy were also excluded from the study.

Data was collected and stored using the Research Electronic Data Capture (REDCap) platform. Collected variables included patient demographics, comorbidities, historical medical attributes, surgical encounter information, and surgical encounter complications.

### Surgical strategy

An incision was made around the peristomal skin around the complete circumference of the ileostomy and carried down through the skin and subcutaneous tissues. The ileostomy was freed from the surrounding subcutaneous structures for the proximal and distal loops of the ileum to be brought extracorporeally. The 2 loops were brought into proximity and a side-to-side, functional end-to-end anastomosis was fashioned with a GIA-75 mm blue load stapler. The common enterotomy was then closed also with GIA-75 mm blue load stapler. The final staple line was oversewn with a 2-0 PDS suture in a running continuous fashion for additional hemostasis. The anastomosis was replaced to the peritoneal cavity. Gowns and gloves were changed prior to closure of fascia with #1 Stratafix suture in a running continuous fashion. The overlying subcutaneous tissues were irrigated and the overlying skin was closed with 0 Vicryl suture in pursestring fashion, leaving a small central opening which was packed with a Betadine soaked Kerlix gauze as a wick.

### Study design

The primary outcome of interest was return of bowel function. Secondary outcomes included any postoperative complications including return to operating room (OR) within the same admission, postoperative anastomotic leak that was confirmed by CT scan, postoperative ileus, which is defined as absence of bowel movements or flatus 3 days post-surgery with symptoms of nausea and/or vomiting, bowel obstruction, postoperative diarrhea, documented nasogastric (NG) tube placement, wound complications, postoperative intra-abdominal abscess, bleeding, anastomotic stenosis, 30-day mortality, hospital length of stay and hospital readmission rate within 30 days. Comparative groups were defined based on the timing of ileostomy reversal: within four months (Control group) or greater than 12 months (Delayed group) after the initial procedure.

### Statistical analysis

Statistical analysis was performed in R Statistical Software (v. 4.3.1). Baseline characteristics and postoperative complications were compared between the two groups. Categorical data were assessed using frequencies, percentages, chi-squared, and Fisher’s exact tests. Numeric data were analyzed with measures of central tendency and variation, along with Wilcoxon rank-sum tests. Statistical modeling included binary logistic regression for 30-day readmissions and a generalized linear modeling for days until bowel function returns with propensity weights to control any confounding data associated treatment group allocation (*p* < 0.02). Propensity weights were calculated using an inverse probability weighting method. Adjusted odds ratios, confidence intervals, and p-values were calculated.

## Results

### Demographics

A total of 636 patients who underwent loop ileostomy reversal were identified within the study period. 135 patients underwent loop ileostomy closure within four months after index operation, and 19 patients underwent closure beyond 12 months after index operation were included in this study. 482 patients who underwent loop ileostomy reversal between four and 12 months were excluded. There were no statistically significant differences in age, sex, body mass index (BMI), percentage of current smokers, history of hypertension, severe chronic obstructive pulmonary disease (COPD), metastatic disease, or inflammatory bowel disease (IBD) between the two groups. The proportion of patients with diabetes (36.84% versus 11.85%, *p* = 0.01), renal failure (26.32% versus 5.93%, *p* = 0.01) and history of colorectal cancer (68.42% versus 32.59%, *p* = 0.003) were higher in the delayed group when compared to the control group. More patients received neoadjuvant or adjuvant chemoradiation in delayed group than controlled group, though not statistically significant (20.00% versus 31.58%, *p* = 0.25) (Table 1).

**Table 1:** Demographics of patients undergoing diverting loop ileostomy closure < 4 months after creation or > 12 months after creation.

	Control	Delayed	*p*-value
	< 4 months	> 12 months	
	*N* = 135	*N* = 19	
Age			0.93
Mean (SD)	55.89 (16.57)	57.53 (11.76)	
Median (IQR)	60.00 (46.50, 67.50)	59.00 (55.50, 62.00)	
Sex			0.20
Female	64 (47.41%)	12 (63.16%)	
Male	71 (52.59%)	7 (36.84%)	
Body Mass Index (BMI)			0.06
Mean (SD)	27.21 (6.01)	28.98 (3.71)	
Median (IQR)	26.00 (23.08, 30.18)	28.94 (27.24, 31.53)	
Current smoker	23 (17.04%)	3 (15.79%)	1.00
Diabetes	16 (11.85%)	7 (36.84%)	0.01
Hypertension	61 (45.19%)	8 (42.11%)	0.80
Severe COPD	12 (8.89%)	1 (5.26%)	1.00
Renal failure	8 (5.93%)	5 (26.32%)	0.01
History and current steroid use	8 (5.93%)	0 (0.00%)	
History and chronic opioid use	10 (7.41%)	2 (10.53%)	0.65
History of colorectal cancer	44 (32.59%)	13 (68.42%)	0.003
History of metastatic disease	7 (5.19%)	2 (10.53%)	0.31
History or current chemo, radiation	27 (20.00%)	6 (31.58%)	0.25
History of IBD	23 (17.04%)	1 (5.26%)	0.31
History of gastroparesis	2 (1.48%)	0 (0.00%)	
History of chronic constipation	5 (3.70%)	1 (5.26%)	0.55

### Postoperative complications

There was no difference in number of days until return of bowel function, which is the primary outcome, between the two groups. Delayed closure was associated with longer operating times (84 min versus 75 min, *p* = 0.04), and more hospital readmission within 30 days when compared to the control group (26.32% versus 8.21%, *p* = 0.03). There was no difference in the total length of stay. Also, there were no statistically significant differences in overall postoperative morbidity, including percentage of patients returned to OR within the same admission, postoperative anastomotic leak, postoperative ileus, bowel obstruction, postoperative diarrhea, wound complications, postoperative intra-abdominal abscess, and bleeding between study groups (Table 2).

**Table 2:** Operative and postoperative outcomes of patients undergoing diverting loop ileostomy closure < 4 months after creation as compared to > 12 months after creation.

	Control	Delayed	*p*-value
	< 4 months	> 12 months	
	*N* = 135	*N* = 19	
Operative time (mins)			0.04
Mean (SD)	87.51 (59.84)	138.53 (129.27)	
Median (IQR)	75.00 (57.00, 99.50)	84.00 (71.00, 120.00)	
Return to OR within the same admission	2 (1.48%)	1 (5.26%)	0.33
Postoperative anastomotic leak	2 (1.48%)	2 (10.53%)	0.08
Postoperative Ileus	11 (8.15%)	4 (21.05%)	0.09
Bowel obstruction	2 (1.48%)	1 (5.26%)	0.33
Postoperative diarrhea	20 (14.81%)	2 (10.53%)	1.00
Documented nasogastric tube placement	11 (8.15%)	3 (15.79%)	0.38
Wound complications	4 (2.96%)	2 (10.53%)	0.16
Postoperative intra-abdominal abscess	4 (2.96%)	1 (5.26%)	0.49
Bleeding	4 (2.96%)	1 (5.26%)	0.49
Anastomotic stenosis	2 (1.49%)	0 (0.00%)	
30-day mortality	0 (0.00%)	0 (0.00%)	
Return of bowel function (days)			0.75
Mean (SD)	2.01 (1.30)	3.05 (4.87)	
Median (IQR)	2.00 (1.00, 2.00)	2.00 (1.00, 2.50)	
Length of stay (days)			0.76
Mean (SD)	3.86 (4.96)	4.98 (5.96)	
Median (IQR)	3.00 (2.00, 4.00)	2.24 (2.04, 3.64)	
Hospital readmission within 30 days	11 (8.21%)	5 (26.32%)	0.03

## Statistical modeling

**Fig. 1:**
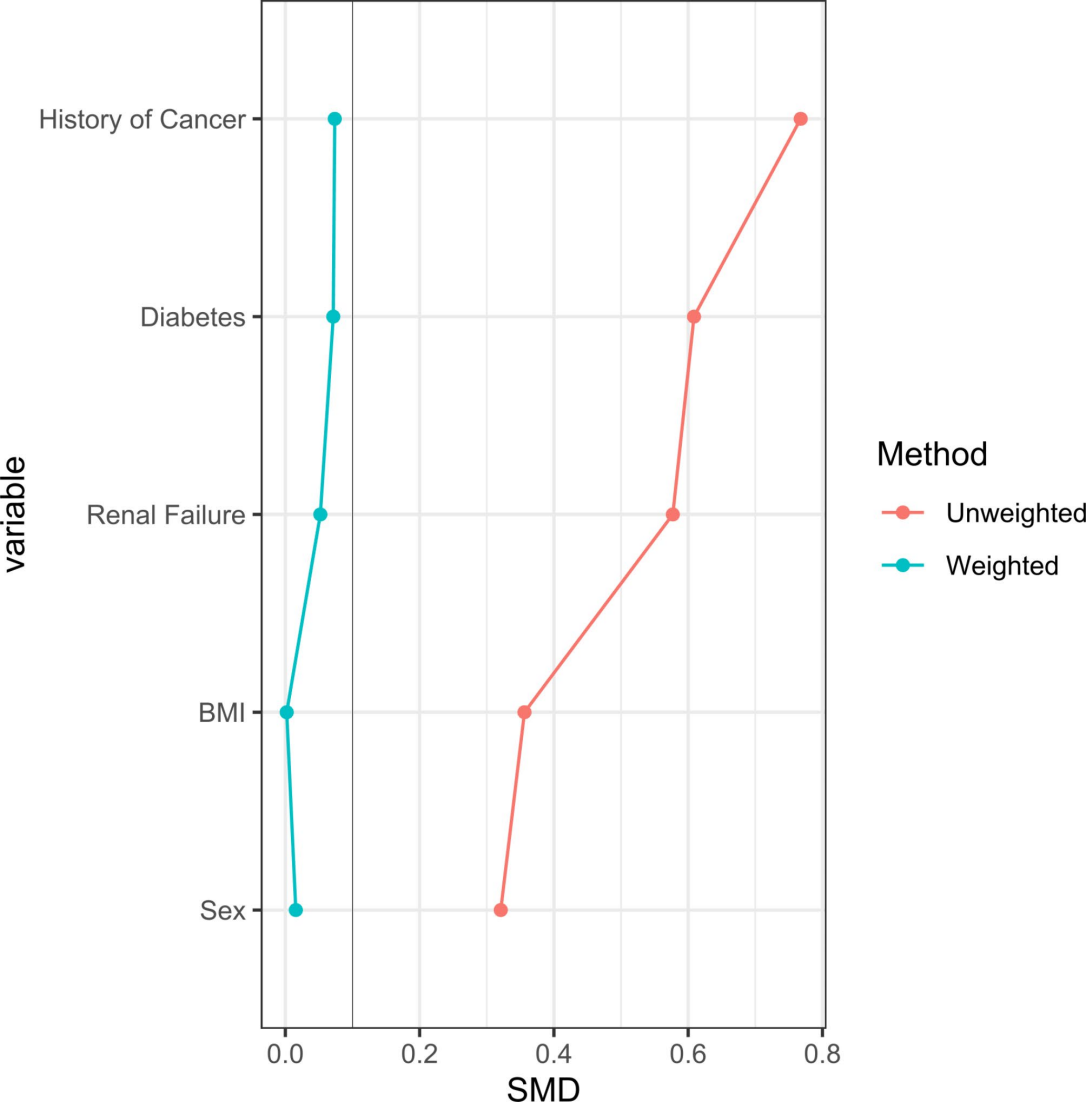
Inverse probability weighting method (IPWM).

An inverse probability weighting method (IPWM) was used to generate weights on sex, BMI, diabetes, renal failure and cancer history, to better estimate differences in postoperative complication rates between the two groups. (Fig. 1) Regression analysis highlighted that hospital readmissions within 30 days remained higher in the delayed group (26.32%) when compared to the control group (8.15%) after weighting. The odds ratio for a readmission occurring in the delayed group was 6.36 (95% C.I. = 3.30, 13.20, *p* < 0.0001). A log transformation was utilized to examine the number of days for bowel function to return in a generalized linear model. The number of days for bowel function to return in the delayed group did not significantly differ from the control group (*p* = 0.56) (Table 3).

**Table 3:** Statistical modeling of primary outcomes with propensity weights.

Statistical modeling of primary outcomes with propensity weights			
Outcome	Observed statistics		Model adjusted P-value
	Delayed group	Control group	
Readmissions	5 (26.32%)	11 (8.15%)	< 0.0001
Return of bowel function	2.00 (1.00, 2.50)	2.00 (1.00, 2.00)	0.56

## Discussion

The timing of loop ileostomy reversal is generally debated among surgeons, and there is no strict consensus^12,13^. However, common practice typically involves reversal within 3 to 6 months after the initial surgery^14^. This timing allows for the healing of the distal anastomosis and minimizes the risks of complications^15^. Timing can vary based on several factors, including the patient’s overall health, the presence of complications, and the specific circumstances of the underlying disease or surgery^14,15^. Early reversal within 3 to 6 months is often pursued to minimize the period of living with an ileostomy and improving the patient’s quality of life. In some patients, reversal could be delayed due to specific patient health considerations, such as the need for additional treatments (e.g., chemotherapy) or recovery from postoperative complications such as anastomotic leak following initial operation or adhesive small bowel obstruction^3,16^. In our study, 19 patients underwent delayed loop ileostomy reversal after 12 months due to complications after their initial surgery, most commonly persistent anastomotic leak or fistula formation. The delayed group had more patients with diabetes and renal failure, both of which are risk factor for poor wound healing leading to fistula formation or anastomotic leak^17–19^. A few patients had delayed reversal due to bowel obstruction and anastomotic stricture after initial surgery. 13 patients had colorectal cancer, representing a statistically significant higher proportion compared to the control group. Among these patients, a higher proportion of patients in the delayed group received adjuvant or neoadjuvant chemoradiation, suggesting that more of these patients had advanced stages of colorectal cancer compared to the control group, and need for delayed reversal due to further cancer treatment.

There was no difference in number of days until return of bowel function, which is the primary endpoint of this study, between the two groups. This showed that while ileostomy reversal could be delayed due to patient and surgical factors, primary outcome and postoperative complications might not be affected by delayed closure. However, the results of this study demonstrate that delayed loop ileostomy closure > 12 months after creation is associated with longer operative times, which emphasizes the complexity and potential challenges encountered during delayed reversal procedures. This is consistent with a prior study that demonstrated that early reversal of loop ileostomy is associated with shorter operative time, which can lead to significant cost savings^20^. To be noted, the average operative time of 138 min for the delayed group is largely due to two outliers in the group that required completion APR and plastic reconstruction for one case and a midline laparotomy incision for extensive lysis of adhesion for the other per chart review. The median operative time is more comparable between the two groups in the setting of extreme outliers. Nevertheless, the delayed group has slightly longer median operative time than the control group likely because more cases required longer lysis of adhesions due to dense adhesions in the delayed group and more cases of parastomal hernia. Prior research has also shown that longer operative times was independently predictive of readmission, which is consistent with the current study finding of higher rates of readmission within 30 days for the delayed group^21^. This highlights the importance of postoperative care and follow-up in this patient cohort.

We acknowledge that propensity weighting may reduce statistical power when investigating readmissions and days to bowel function. Therefore, unadjusted p-values have also been reported to provide a more powered assessment. Although the sample sizes for each group are small, several covariates were found to be unbalanced between the two groups. Propensity weighting demonstrated more balanced comparison groups.

While numerous studies have shown that delayed loop ileostomy reversal results in dysfunctional digestive problems—either nausea or vomiting; or ileus, diarrhea or stool incontinence^5,8,10,22–24^, our analysis did not unveil significant differences in the days of return of bowel function, postoperative ileus or postoperative obstruction, even after weighting and regression analysis. However, postoperative leak and ileus were marginally insignificant likely due to the small sample size of this study. Since the groups were not matched in terms of comorbidities or postoperative complications after the index loop ileostomy creation operation, someone could assume that the highest rate in leak, ileus and readmission could be attributed to those factors and not in the delayed closure.

This study did not show differences in wound complications and bleeding between control and delayed group, which is consistent with a pre-existing retrospective study comparing outcomes between patients who had early (< 6 months) or late (> 6 months) loop ileostomy closure showing no difference in wound complications and bleeding^23^. This study found no significant differences in the incidence of postoperative intra-abdominal abscess, anastomotic stenosis, or postoperative anastomotic leak between the delayed and control groups. These findings contribute to the existing knowledge by suggesting that the relationship between delayed ileostomy reversal and these specific complications remains inconclusive. This underscores the need for further research to definitively establish the risks associated with delayed ileostomy reversal.

The study has several limitations that should be acknowledged. The small sample size, particularly in the delayed reversal group, may have limited the statistical power to detect significant differences in complications. The retrospective design introduces the potential for selection bias, as data were collected from past records instead of a prospective, randomized trial. Furthermore, there were confounding factors, such as varying stages of colorectal cancer and the receipt of adjuvant or neoadjuvant chemoradiation, which could have influenced postoperative outcomes like operative time and complications. Differences in baseline comorbidities, such as higher rates of diabetes and renal failure in the delayed group, also impacted results, particularly regarding fistula formation. Additionally, the longer operative times in the delayed group were driven by a few outliers involving complex cases, which may not represent typical delayed reversals. Despite propensity weighting to balance groups, residual imbalances may still exist due to the cohort’s small size and multiple comorbidities. Lastly, being a single-center study, the findings may not be generalizable to other institutions or patient populations. These limitations highlight the need for larger, prospective studies to clarify the optimal timing and outcomes of loop ileostomy reversal.

For the future direction of this ileostomy study, investigating the role of butyrylcholinesterase (BChE) levels as a predictive marker for postoperative outcomes could provide valuable insight. BChE is known to reflect a patient’s nutritional and metabolic status, and low levels have been linked to poor surgical outcomes, including increased risk of complications such as infections, anastomotic leaks, and delayed recovery. Given that delayed loop ileostomy reversal often involves patients with significant comorbidities or complications post-initial surgery, such as diabetes or renal failure, preoperative BChE levels could be used to identify high-risk patients who may require more intensive perioperative care. Future studies could focus on assessing BChE levels in patients undergoing both early and delayed ileostomy reversals to determine if this marker correlates with increased rates of postoperative complications, length of hospital stay, or delayed bowel function. Furthermore, integrating BChE levels with other known predictors of postoperative risk, such as albumin levels, CRP, and patient comorbidities, could refine risk stratification models and help guide individualized surgical planning and postoperative management^25,26^.

## Conclusions

In conclusion, our study demonstrates that delayed ileostomy closure more than 12 months after initial operation might result in longer operative times and higher 30-day readmission, but did not result in a difference in time to return of bowel function. This underscores the importance of individualized decision-making in loop ileostomy closure, considering patient-specific factors and the potential trade-offs associated with timing. As the field continues to evolve, ongoing research endeavors will be vital in refining guidelines and optimizing outcomes for patients undergoing this common aspect of colorectal surgery.

## Data Availability

Data is provided within the manuscript and stored in our institution’s RedCap server. The datasets used and/or analysed during the current study are available from the corresponding author on reasonable request.
